# Predictors of relapse in bipolar disorder: an overview of the available evidence

**DOI:** 10.1192/j.eurpsy.2024.113

**Published:** 2024-08-27

**Authors:** I. Pacchiarotti

**Affiliations:** ^1^Institute of Neurosciences, Hospital Clínic of Barcelona; ^2^Bipolar and Depressive Disorders Unit, IDIBAPS, Barcelona, Spain

## Abstract

**Abstract:**

After the introduction of all the speakers, the main aim of this workshop will be mentioned, which consists of identifying and highlighting those clinical, sociodemographic, environmental and other factors than might predict an increased risk of overall, depressive, manic or mixed relapses in bipolar disorder, which is crucial for the identification of high-risk individuals. Dr. Pacchiarotti will present main results from a systematic review performed recently by the work group aimed at collecting the available evidence regarding different factors that increase rates of mood recurrences or relapses for different polarities in bipolar disorder.

**Disclosure of Interest:**

None Declared

